# Supplementary material for: Participation bias in the estimation of heritability and genetic correlation
Source: Proc Natl Acad Sci U S A. 2025 Jun 20;122(25):e2425530122. doi: 10.1073/pnas.2425530122 (PMC12207467; doi:10.1073/pnas.2425530122)
Supplement: Supplementary file 1 — Appendix 01 (PDF) [file pnas.2425530122.sapp.pdf]

Supporting Information for

Participation bias in the estimation of heritability and genetic correlation

Shuang Song, Stefania Benonisdottir, Jun S. Liu and Augustine Kong

Shuang Song, Augustine Kong.
E-mail: shuangsong@hsph.harvard.edu, augustine.kong@bdi.ox.ac.uk

This PDF file includes:

Supporting text
Fig. S1
Tables S1 to S4
SI References

Supporting Information Text

A. Conditional correlation of truncated normal distribution. We first provide some results on the conditional variance and covariance of truncated normal distribution, and then derive the conditional correlation. Consider three normally distributed variables:

$$\begin{pmatrix} Z_1 \\ Z_2 \\ Z_3 \end{pmatrix} \sim \mathcal{N}\left(\begin{pmatrix} \mu_1 \\ \mu_2 \\ \mu_3 \end{pmatrix}, \Sigma^{\frac{1}{2}} \begin{pmatrix} 1 & \rho_{12} & \rho_{13} \\ \rho_{12} & 1 & \rho_{23} \\ \rho_{13} & \rho_{23} & 1 \end{pmatrix} \Sigma^{\frac{1}{2}} \right), \quad [1]$$

where $\Sigma = \mathrm{diag}(\sigma_1^2, \sigma_2^2, \sigma_3^2)$, and ρ_{kl} are the correlation between Z_k and Z_l. We first derive the variance of Z_1 conditioning on $Z_3 \geq c$ under a simplified case when $\mu_1 = \mu_2 = \mu_3 = 0$, and $\sigma_1^2 = \sigma_2^2 = \sigma_3^2 = 1$, where c is a certain threshold. From Kan and Robotti (2016) (1), we have

$$\mathbb{E}(Z_3 | Z_3 \geq c) = \frac{\phi(c)}{\Phi(-c)}, \quad [2]$$

where $\phi(\cdot)$ is the standard normal probability density function (PDF), and $\Phi(\cdot)$ is the standard normal cumulative distribution function (CDF). In addition, we have

$$\mathrm{Var}(Z_3 | Z_3 \geq c) = 1 + c \cdot \frac{\phi(c)}{\Phi(-c)} - \left[\frac{\phi(c)}{\Phi(-c)} \right]^2. \quad [3]$$

For simplicity in notation, we let

$$\tau(c) = c \cdot \frac{\phi(c)}{\Phi(-c)} - \left[\frac{\phi(c)}{\Phi(-c)} \right]^2. \quad [4]$$

Then we have

$$\begin{aligned} &\mathrm{Var}(Z_1 | Z_3 \geq c) = \mathbb{E}(Z_1^2 | Z_3 \geq c) - \mathbb{E}(Z_1 | Z_3 \geq c)^2 \\ =& \int_c^\infty \mathbb{E}(Z_1^2 | z_3) f_{Z_3|Z_3 \geq c}(z_3) dz_3 - \left(\int_c^\infty \mathbb{E}(Z_1 | z_3) f_{Z_3|Z_3 \geq c}(z_3) dz_3 \right)^2 \\ =& \int_c^\infty \left[\rho_{13}^2 Z_3^2 + (1 - \rho_{13}^2) \right] f_{Z_3|Z_3 \geq c}(z_3) dz_3 - \left(\int_c^\infty \rho_{13} Z_3 f_{Z_3|Z_3 \geq c}(z_3) dz_3 \right)^2 \\ =& \rho_{13}^2 \cdot \mathbb{E}(Z_3^2 | Z_3 \geq c) + (1 - \rho_{13}^2) - \rho_{13}^2 \cdot \mathbb{E}(Z_3 | Z_3 \geq c)^2 \\ =& 1 - \rho_{13}^2 + \rho_{13}^2 \cdot \mathrm{Var}(Z_3 | Z_3 \geq c) \\ =& 1 + \rho_{13}^2 \cdot \tau(c), \end{aligned} \quad [5]$$

where $f_{Z_3|Z_3 \geq c}(\cdot)$ denotes the conditional PDF of Z_3 given $\{Z_3 \geq c\}$, and ρ_{ij} denotes the correlation between Z_i and Z_j. Then we can derive the general case when Z_1 and Z_3 have nonzero mean μ_1 and μ_3, and variance σ_1^2 and σ_3^2:

$$\mathrm{Var}(Z_1 | Z_3 \geq c) = \sigma_1^2 \left[1 + \rho_{13}^2 \cdot \tau\left(\frac{c + \mu_3}{\sigma_3} \right) \right], \quad [6]$$

Similarly, we have

$$\mathrm{Var}(Z_2 | Z_3 \geq c) = \sigma_2^2 \left[1 + \rho_{23}^2 \cdot \tau\left(\frac{c + \mu_3}{\sigma_3} \right) \right]. \quad [7]$$

We further focus on the covariance between Z_1 and Z_2 conditioning on $Z_3 \geq c$. When $\mu_1 = \mu_2 = \mu_3 = 0$ and $\sigma_1^2 = \sigma_2^2 = \sigma_3^2 = 1$, from the properties of truncated normal distribution, we have

$$\begin{aligned} &\mathrm{Cov}(Z_1, Z_2 | Z_3 \geq c) = \mathbb{E}(Z_1 Z_2 | Z_3 \geq c) - \mathbb{E}(Z_1 | Z_3 \geq c) \cdot \mathbb{E}(Z_2 | Z_3 \geq c) \\ =& \int_c^\infty \mathbb{E}(Z_1 Z_2 | z_3) f_{Z_3|Z_3 \geq c}(z_3) dz_3 - \int_c^\infty \mathbb{E}(Z_1 | z_3) f_{Z_3|Z_3 \geq c}(z_3) dz_3 \cdot \int_c^\infty \mathbb{E}(Z_2 | z_3) f_{Z_3|Z_3 \geq c}(z_3) dz_3 \\ =& \int_c^\infty \left[\mathrm{Cov}(Z_1, Z_2 | z_3) + \mathbb{E}(Z_1 | z_3) \cdot \mathbb{E}(Z_2 | z_3) \right] f_{Z_3|Z_3 \geq c}(z_3) dz_3 - \rho_{13}\rho_{23} \left[\mathbb{E}(Z_3 | Z_3 \geq c) \right]^2 \\ =& \rho_{12} - \rho_{13}\rho_{23} + \rho_{13}\rho_{23} \cdot \mathbb{E}(Z_3^2 | Z_3 \geq c) - \rho_{13}\rho_{23} \cdot \left[\mathbb{E}(Z_3 | Z_3 \geq c) \right]^2 \\ =& \rho_{12} - \rho_{13}\rho_{23} + \rho_{13}\rho_{23} \cdot \mathrm{Var}(Z_3 | Z_3 \geq c) \\ =& \rho_{12} + \rho_{13}\rho_{23} \cdot \tau(c). \end{aligned} \quad [8]$$

The results under the general case when Z_1, Z_2, and Z_3 have nonzero mean μ_1, μ_2, and μ_3, and variance σ_1^2, σ_2^2, and σ_3^2 would be:

$$\mathrm{Cov}(Z_1, Z_2 | Z_3 \geq c) = \quad \sigma_1 \sigma_2 \left[\rho_{12} + \rho_{13}\rho_{23} \cdot \tau\left(\frac{c + \mu_3}{\sigma_3} \right) \right]. \quad [9]$$

Combining Eq. (6), Eq. (7), and Eq. (9), we have

$$\begin{aligned}\mathrm{Corr}(Z_1, Z_2|Z_3 \geq c) &= \frac{\mathrm{Cov}(Z_1, Z_2|Z_3 \geq c)}{\sqrt{\mathrm{Var}(Z_1|Z_3 \geq c)}\sqrt{\mathrm{Var}(Z_2|Z_3 \geq c)}} \\ &= \frac{\rho_{12} + \rho_{13}\rho_{23} \cdot \tau\left(\frac{c+\mu_3}{\sigma_3}\right)}{\sqrt{\left[1 + \rho_{13}^2 \cdot \tau\left(\frac{c+\mu_3}{\sigma_3}\right)\right]\left[1 + \rho_{23}^2 \cdot \tau\left(\frac{c+\mu_3}{\sigma_3}\right)\right]}}.\end{aligned} \qquad [10]$$

B. Heritability estimated with participation bias. Under the model (1) described in the main text, the heritability in the population is defined as $h_y^2 = \mathrm{Corr}(Y, G_y)^2$. The genetic component G_y can be reparametrized as $a \cdot G_x + G_w$, where G_x and G_w are independent. Specifically, $\mathrm{Var}(G_x) = h_x^2$, and $\mathrm{Var}(G_y) = h_y^2$, and thus $a = \mathrm{Corr}(G_x, G_y) \cdot \sqrt{\mathrm{Var}(G_y)/\mathrm{Var}(G_x)} = \rho_G/h_x^2 = \rho_g\sqrt{h_y^2/h_x^2}$. Similarly, we decompose the non-genetic component of Y with $\epsilon_y = b \cdot \epsilon_x + \epsilon_w$, where $b = \rho_E/(1 - h_x^2) = \rho_e\sqrt{(1 - h_y^2)/(1 - h_x^2)}$. We assume the genetic and non-genetic components (G_x, G_w, ϵ_x, ϵ_w) jointly follow a multivariate normal distribution with a mean vector of zero and covariance matrix diag $\left[h_x^2, h_y^2(1 - \rho_g^2), 1 - h_x^2, (1 - h_y^2) \cdot (1 - \rho_e^2)\right]$. We can therefore compute the covariance matrix of (X, Y, G_x, G_w):

$$\begin{pmatrix} 1 & \rho & h_x^2 & 0 \\ \rho & 1 & \rho_G & h_y^2(1-\rho_g^2) \\ h_x^2 & \rho_G & h_x^2 & 0 \\ 0 & h_y^2(1-\rho_g^2) & 0 & h_y^2(1-\rho_g^2) \end{pmatrix}. \qquad [11]$$

Intuitively, $h_{y,PB}^2$ represents the proportion of variance explained by genetic components in the sample of participants, which is the "apparent" heritability that would be estimated if participation bias (PB) is ignored. As shown in Figure 1 of the main context, the genetic component of Y in the sample of participants can also be decomposed into G_x and G_w. The coefficient of G_w remains unchanged, as both G_x and e_x are independent of G_w. However, the coefficient of G_x, denoted by a', is smaller than a because G_x and ϵ_x become negatively correlated in the sample of participants, and the negative correlation is absorbed into the genetic component. We use $\mathcal{P}$ to denote participation, *i.e.* $X > t_\alpha$. The coefficient of G_x can be computed as:

$$a' = a + b \cdot \frac{\mathrm{Cov}(G_x, \epsilon_x|\mathcal{P})}{\mathrm{Var}(G_x|\mathcal{P})} = a - b \cdot \frac{\xi(\alpha) \cdot h_x^2(1 - h_x^2)}{h_x^2\left[1 - \xi(\alpha)h_x^2\right]} = \frac{\rho_G}{h_x^2} + \frac{\xi(\alpha)\rho_E}{1 - \xi(\alpha)h_x^2}. \qquad [12]$$

We use G_y' to denote the genetic component of Y in the sample of participants, and we show that with the decomposition $G_y' = a' \cdot G_x + G_w$, G_y' is orthogonal to the non-genetic components in the sample of participants:

$$\begin{aligned}\mathrm{Cov}(G_y', Y - G_y'|\mathcal{P}) &= \mathrm{Cov}(a' \cdot G_x + G_w, (a - a') \cdot G_x - \epsilon_y|\mathcal{P}) \\ &= a'\left[(a - a') \cdot \mathrm{Var}(G_x|\mathcal{P}) - \mathrm{Cov}(G_x, \epsilon_y|\mathcal{P})\right] \\ &= a'\left[\xi(\alpha)\rho_E h_x^2 - \xi(\alpha)\rho_E h_x^2\right] = 0.\end{aligned} \qquad [13]$$

The heritability of Y in the sample of participants is calculated with:

$$\begin{aligned}h_{y,PB}^2 = \frac{\mathrm{Var}(G_y'|\mathcal{P})}{\mathrm{Var}(Y|\mathcal{P})} &= \frac{a'^2 \cdot \mathrm{Var}(G_x|\mathcal{P}) + \mathrm{Var}(G_w|\mathcal{P})}{1 - \xi(\alpha)\rho^2} \\ &= \frac{1}{1 - \xi(\alpha)\rho^2} \cdot \left[\frac{(\rho_G - \xi(\alpha)\rho h_x^2)^2}{h_x^2(1 - \xi(\alpha)h_x^2)} + h_y^2 - \frac{\rho_G^2}{h_x^2}\right] \\ &= \frac{1}{1 - \xi(\alpha)\rho^2} \cdot \left[h_y^2 + \frac{(\rho_G - \xi(\alpha)\rho h_x^2)^2 - \rho_G^2(1 - \xi(\alpha)h_x^2)}{h_x^2(1 - \xi(\alpha)h_x^2)}\right] \\ &= \frac{1}{1 - \xi(\alpha)\rho^2} \cdot \left[h_y^2 + \frac{-2\rho_G\xi(\alpha)\rho h_x^2 + (\xi(\alpha)\rho h_x^2)^2 + \xi(\alpha)\rho_G^2 h_x^2}{h_x^2(1 - \xi(\alpha)h_x^2)}\right] \\ &= \frac{1}{1 - \xi(\alpha)\rho^2} \cdot \left[h_y^2 - \xi(\alpha)\rho_G(\rho_G + 2\rho_E) + \frac{\xi(\alpha)^2\rho_E^2 h_x^2}{1 - \xi(\alpha)h_x^2}\right].\end{aligned} \qquad [14]$$

C. Genetic correlation underlying participation bias. We consider two related traits, Y_1 and Y_2, following the additive model:

$$\begin{aligned}Y_1 &= G_{y_1} + \epsilon_{y_1}, \\ Y_2 &= G_{y_2} + \epsilon_{y_2},\end{aligned} \qquad [15]$$

where both Y_1 and Y_2 are standardized. We use φ_g to denote the correlation between G_{y_1} and G_{y_2} in the population. We consider the decomposition $G_{y_1} = a_1 \cdot G_x + G_{w_1}$ and $G_{y_2} = a_2 \cdot G_x + G_{w_2}$, where $a_1 = \rho_{G_1}/h_x^2$ and $a_2 = \rho_{G_2}/h_x^2$, ensuring G_x to be independent to G_{w_1} and G_{w_2}. We use G_{y_1}' and G_{y_2}' to denote the genetic component in the sample of participants, which can be decomposed as $G_{y_1}' = a_1' \cdot G_x + G_{w_1}$, and $G_{y_2}' = a_2' \cdot G_x + G_{w_2}$, where $a_l' = \rho_{G_l}/h_x^2 - \xi(\alpha)\rho_{E_l}/\left[1 - \xi(\alpha)h_x^2\right]$, $l = 1, 2$.

Here we define the genetic covariance as the covariance between genetic components with standardized phenotypes. Thus, the genetic covariance between Y_1 and Y_2 in the sample of participants is defined as

$$\varphi_{G,PB} = \frac{\mathrm{Cov}(G'_{y_1}, G'_{y_2}|\mathcal{P})}{\sqrt{\mathrm{Var}(Y_1|\mathcal{P}) \cdot \mathrm{Var}(Y_2|\mathcal{P})}}, \tag{16}$$

which is the "apparent" genetic covariance of Y_1 and Y_2 that would be estimated is PB is ignored in the sample of participants. The corresponding correlation is $\varphi_{g,PB} = \mathrm{Corr}(G'_{y_1}, G'_{y_2}|\mathcal{P})$. Based on Eq. (6) and Eq. (9), we have

$$\begin{aligned}\varphi_{G,PB} &= \frac{\mathrm{Cov}(G'_{y_1}, G'_{y_2}|\mathcal{P})}{\sqrt{\mathrm{Var}(Y_1|\mathcal{P}) \cdot \mathrm{Var}(Y_2|\mathcal{P})}} = \frac{\mathrm{Cov}(a'_1 \cdot G_x + G_{w_1}, a'_2 \cdot G_x + G_{w_2}|\mathcal{P})}{\sqrt{(1-\xi(\alpha)\rho_1^2)\cdot(1-\xi(\alpha)\rho_2^2)}} \\ &= \frac{1}{\sqrt{(1-\xi(\alpha)\rho_1^2)\cdot(1-\xi(\alpha)\rho_2^2)}}\left[a'_1 a'_2 \cdot \mathrm{Var}(G_x|\mathcal{P}) + \mathrm{Cov}(G_{w_1}, G_{w_2})\right] \\ &= \frac{1}{\sqrt{(1-\xi(\alpha)\rho_1^2)\cdot(1-\xi(\alpha)\rho_2^2)}}\left[\frac{(\rho_{G_1} - \xi(\alpha)\rho_1 h_x^2)\cdot(\rho_{G_2} - \xi(\alpha)\rho_2 h_x^2)}{h_x^2(1-\xi(\alpha)h_x^2)} + \varphi_G - \frac{\rho_{G_1}\rho_{G_2}}{h_x^2}\right] \\ &= \frac{1}{\sqrt{(1-\xi(\alpha)\rho_1^2)\cdot(1-\xi(\alpha)\rho_2^2)}}\left[\varphi_G - \xi(\alpha)(\rho_{E_1}\rho_{G_2} + \rho_{E_2}\rho_{G_1} + \rho_{G_1}\rho_{G_2}) + \frac{\xi(\alpha)^2\rho_{E_1}\rho_{E_2}h_x^2}{1-\xi(\alpha)h_x^2}\right].\end{aligned} \tag{17}$$

The third equality arises because G_x and G_{w_l} are independent after selection. In addition, the conditional variances of G_{w_l} remain unchanged.

We further compute the corresponding genetic correlation:

$$\varphi_{g,PB} = \frac{\varphi_{G,PB}}{\sqrt{h^2_{y_1,PB} \cdot h^2_{y_2,PB}}} = \frac{\varphi_G - \xi(\alpha)(\rho_{E_1}\rho_{G_2} + \rho_{E_2}\rho_{G_1} + \rho_{G_1}\rho_{G_2}) + \frac{\xi(\alpha)^2\rho_{E_1}\rho_{E_2}h_x^2}{1-\xi(\alpha)h_x^2}}{\sqrt{(1-\xi(\alpha)\rho_1^2)\cdot(1-\xi(\alpha)\rho_2^2)\cdot h^2_{y_1,PB}\cdot h^2_{y_2,PB}}}, \tag{18}$$

where $h^2_{y_1,PB}$ and $h^2_{y_2,PB}$ are derived in Eq. (14).

D. Genetic correlation between participation and a phenotype. The liability score underlying participation can be thought of as a special phenotype. Similar to Section C, we define the genetic covariance as the covariance between genetic components with standardized phenotypes. Specifically, we consider G_x in the population and G'_y in the selected sample, and the genetic covariance is defined as

$$\rho_{G,PB} = \frac{\mathrm{Cov}(G_x, G'_y|\mathcal{P})}{\sqrt{\mathrm{Var}(Y|\mathcal{P})}}. \tag{19}$$

The corresponding genetic correlation is $\rho_{g,PB} = \mathrm{Corr}(G_x, G'_y|\mathcal{P})$. Note that G_x inside the expression of $\rho_{G,PB}$ and $\rho_{g,PB}$ is the genetic component of X in the population, which is not affected by PB.

Based on Eq. (6) and Eq. (9), we have

$$\begin{aligned}\rho_{G,PB} &= \frac{\mathrm{Cov}(G_x, G'_y|\mathcal{P})}{\sqrt{\mathrm{Var}(Y|\mathcal{P})}} = \frac{\mathrm{Cov}(G_x, a'\cdot G_x + G_w|\mathcal{P})}{\sqrt{1-\xi(\alpha)\rho^2}} = \frac{a'\cdot\mathrm{Var}(G_x|\mathcal{P})}{\sqrt{1-\xi(\alpha)\rho^2}} \\ &= \frac{1}{\sqrt{1-\xi(\alpha)\rho^2}}\left(\frac{\rho_G}{h_x^2} - \frac{\xi(\alpha)\rho_E}{1-\xi(\alpha)h_x^2}\right)\cdot\left[h_x^2(1-\xi(\alpha)h_x^2)\right] \\ &= \frac{1}{\sqrt{1-\xi(\alpha)\rho^2}}\left(\rho_G - \xi(\alpha)h_x^2\rho\right).\end{aligned} \tag{20}$$

We further derive the genetic correlation between participation and the phenotype:

$$\begin{aligned}\rho_{g,PB} = \mathrm{Corr}(G_x, G'_y|\mathcal{P}) &= \frac{\mathrm{Cov}(G_x, G'_y|\mathcal{P})}{\sqrt{\mathrm{Var}(G_x|\mathcal{P})\cdot\mathrm{Var}(G'_y|\mathcal{P})}} = \frac{\mathrm{Cov}(G_x, G'_y|\mathcal{P})}{\sqrt{\mathrm{Var}(G_x|\mathcal{P})\cdot\mathrm{Var}(Y|\mathcal{P})\cdot h^2_{y,PB}}} \\ &= \frac{\rho_{G,PB}}{\sqrt{(1-\xi(\alpha)h_x^2)\cdot h_x^2\cdot h^2_{y,PB}}} = \frac{\rho_G - \xi(\alpha)\rho h_x^2}{\sqrt{(1-\xi(\alpha)\rho^2)\cdot(1-\xi(\alpha)h_x^2)\cdot h_x^2\cdot h^2_{y,PB}}}.\end{aligned} \tag{21}$$

E. LDSC estimates underlying participation bias.

E.1. Heritability. LDSC considers an additive model:

$$X = \boldsymbol{G}\boldsymbol{\beta} + \epsilon_x,$$
$$Y = \boldsymbol{G}\boldsymbol{\gamma} + \epsilon_y, \qquad [22]$$

where $\boldsymbol{G}$ is the standardized genotypic vector, and $\boldsymbol{\beta}$ and $\boldsymbol{\gamma}$ are the effect sizes. Specifically, they assume the effect sizes in the population are normally distributed as $\boldsymbol{\beta} \sim N(0, \frac{h_x^2}{p}\mathbf{I})$ and $\boldsymbol{\gamma} \sim N(0, \frac{h_y^2}{p}\mathbf{I})$, where p is the number of SNPs (2). They further assume the genetic effects on the participation liability score X and related trait Y have the covariance structure:

$$\mathrm{Cov}(\boldsymbol{\beta}, \boldsymbol{\gamma}) = \frac{1}{p}\begin{pmatrix} h_x^2 & \rho_g\sqrt{h_x^2 h_y^2} \\ \rho_g\sqrt{h_x^2 h_y^2} & h_y^2 \end{pmatrix} \otimes \mathbf{I}. \qquad [23]$$

With the above assumptions, LDSC provides the unbiased estimate of h_y^2 in the population.

Now we consider the LDSC estimates in the sample of participants. We assume the genotypic and phenotypic values can be approximated by a multivariate normal distribution when the number of variants affecting the trait is large enough (3). We indicate the approximation with the $\simeq$ sign. We denote the genotype of the j-th SNP as g_j, and the per-standardized marginal genetic effect of the phenotype in the sample of participants as $\boldsymbol{\gamma}'^*$, and the per-standardized marginal genetic effect of participation in the population as $\boldsymbol{\beta}^*$. For the j-th SNP, we have

$$\gamma_j'^* = \mathrm{Corr}(Y, g_j|\mathcal{P}) \simeq \frac{\gamma_j^* - \xi(\alpha)\rho\beta_j^*}{\sqrt{(1-\xi(\alpha)\rho^2)(1-\xi(\alpha)\beta_j^{*2})}}, \qquad [24]$$

and we have

$$\boldsymbol{\gamma}'^* \simeq \frac{1}{\sqrt{1-\xi(\alpha)\rho^2}} \cdot \mathrm{diag}(\frac{1}{\sqrt{1-\xi(\alpha)\beta_j^{*2}}}) \cdot \boldsymbol{R} \cdot (\boldsymbol{\gamma} - \xi(\alpha)\rho\boldsymbol{\beta}), \qquad [25]$$

where $\boldsymbol{R}$ is the LD matrix in the population.

In the sample of participants, we denote the observed per-standardized marginal effect size of the j-th SNP as $\gamma_{j,sam}^*$, and we have $\gamma_{j,sam}^* = \gamma_j'^* + \zeta_j$, where $\mathbb{E}(\zeta_j) = 0$, and $\mathrm{Var}(\zeta_j) \approx 1/N_{sam}$ when $p \to \infty$. Conditioning on $\boldsymbol{\beta}$ and $\boldsymbol{\gamma}$, we have

$$\mathbb{E}(\gamma_{j,sam}^{*2}|\boldsymbol{\beta}, \boldsymbol{\gamma}) = \mathbb{E}(\gamma_j'^{*2} + 2\gamma_j'^* \cdot \zeta_j + \zeta_j^2|\boldsymbol{\beta}, \boldsymbol{\gamma}) = \gamma_j'^{*2} + \mathbb{E}(\zeta_j^2) \approx \gamma_j'^{*2} + \frac{1}{N_{sam}}. \qquad [26]$$

From Eq. (25), we have

$$\begin{aligned}\mathbb{E}(\gamma_{j,sam}^{*2}) &= \mathbb{E}\left[\mathbb{E}(\gamma_{j,sam}^{*2}|\boldsymbol{\gamma}, \boldsymbol{\beta})\right] \approx \mathbb{E}\left[\frac{(\gamma_j^* - \xi(\alpha)\rho\beta_j^*)^2}{(1-\rho^2\xi(\alpha))(1-\xi(\alpha)\beta_j^{*2})}\right] + \frac{1}{N_{sam}} \\ &= \mathbb{E}\left\{\frac{\left[\sum_{k=1}^p r_{kj}(\gamma_k - \xi(\alpha)\rho\beta_k)\right]^2}{(1-\xi(\alpha)\rho^2)\cdot\left[1-\xi(\alpha)(\sum_{k=1}^p r_{kj}\beta_k)^2\right]}\right\} + \frac{1}{N_{sam}} \\ &\approx \frac{h_y^2 - 2\xi(\alpha)\rho\rho_G + \xi(\alpha)^2\rho^2 h_x^2}{p(1-\xi(\alpha)\rho^2)} l_j + \frac{1}{N_{sam}}.\end{aligned} \qquad [27]$$

We assume the C-dependent structure to the SNPs in the population (i.e., SNPs with distance larger than C are independent), and C is $\mathcal{O}(1)$, which is a resonable assumption as discussed in Jiang et al., (2021) (4). Then the second approximation hides $\mathcal{O}(1/p)$ error from disregarding the term of $\xi(\alpha)(\sum_{k=1}^p r_{kj}\beta_k)^2$ in the denominator. It follows that the expected χ^2-statistic observed at variant j in the sample of participants is:

$$\mathbb{E}(\chi_j^2) = \frac{\mathbb{E}(\gamma_{j,sam}^{*2})}{1/N_{sam}} \approx \frac{N_{sam}(h_y^2 - 2\xi(\alpha)\rho\rho_G + \xi(\alpha)^2\rho^2 h_x^2)}{p(1-\xi(\alpha)\rho^2)} l_j + 1. \qquad [28]$$

Thus, when $p \to \infty$, the LDSC estimates of heritability is

$$\mathbb{E}(\widehat{h}_{y,LDSC}^2) = \frac{h_y^2 - 2\xi(\alpha)\rho\rho_G + \xi(\alpha)^2\rho^2 h_x^2}{1-\xi(\alpha)\rho^2}. \qquad [29]$$

E.2. Genetic covariance across two related traits. We further consider the LDSC estimate of genetic correlation between two related traits in the sample of participants, ignoring PB. Similarly, for the j-th SNP, we have the observed marginal effect in the sample of participants $\gamma_{1j,sam}^* = \gamma_{1j}'^* + \zeta_{1j}$; and $\gamma_{2j,sam}^* = \gamma_{2j}'^* + \zeta_{2j}$. The errors have $\mathbb{E}(\zeta_{1j}) = \mathbb{E}(\zeta_{2j}) = 0$; $\mathrm{Var}(\zeta_{1j}) \approx 1/N_{1,sam}$ and $\mathrm{Var}(\zeta_{2j}) \approx 1/N_{2,sam}$ when $p \to \infty$. We denote the number of shared samples in two studies is N_s. Then we have

$$\begin{aligned}\mathbb{E}(\gamma_{1j,sam}^*\gamma_{2j,sam}^*|\boldsymbol{\beta}, \boldsymbol{\gamma}_1, \boldsymbol{\gamma}_2) &= \mathbb{E}\left[(\gamma_{1j}'^*\gamma_{2j}'^* + \gamma_{1j}'^* \cdot \zeta_{2j} + \gamma_{2j}'^* \cdot \zeta_{1j} + \zeta_{1j}\zeta_{2j})|\boldsymbol{\beta}, \boldsymbol{\gamma}_1, \boldsymbol{\gamma}_2\right] \\ &= \gamma_{1j}'^*\gamma_{2j}'^* + \mathbb{E}(\zeta_{1j}\zeta_{2j}) \approx \gamma_{1j}'^*\gamma_{2j}'^* + \frac{N_s\varphi'}{N_{1,sam}N_{2,sam}},\end{aligned} \qquad [30]$$

where φ' is the phenotypic correlation of the two trait in the sample of participants. From Eq. (25), we have

$$\begin{aligned}
\mathbb{E}(\gamma^*_{1j,sam}\gamma^*_{2j,sam}) &= \mathbb{E}\left[\mathbb{E}(\gamma^*_{1j,sam}\gamma^*_{2j,sam}|\boldsymbol{\beta},\boldsymbol{\gamma}_1,\boldsymbol{\gamma}_2)\right] \\
&\approx \mathbb{E}\left[\frac{(\gamma^*_{1j}-\xi(\alpha)\rho_1\beta^*_j)(\gamma^*_{2j}-\xi(\alpha)\rho_2\beta^*_j)}{\sqrt{(1-\xi(\alpha)\rho_1^2)(1-\xi(\alpha)\rho_2^2)}\cdot(1-\xi(\alpha)\beta^{*2}_j)}\right]+\frac{N_s\varphi'}{N_{1,sam}N_{2,sam}} \\
&= \mathbb{E}\left\{\frac{\left[\sum_{k=1}^p r_{kj}(\gamma_{1k}-\xi(\alpha)\rho_1\beta_k)\right]\cdot\left[\sum_{k=1}^p r_{kj}(\gamma_{2k}-\xi(\alpha)\rho_2\beta_k)\right]}{\sqrt{(1-\xi(\alpha)\rho_1^2)(1-\xi(\alpha)\rho_2^2)}\cdot\left[1-\xi(\alpha)(\sum_{k=1}^p r_{kj}\beta_k)^2\right]}\right\}+\frac{N_s\varphi'}{N_{1,sam}N_{2,sam}} \\
&\approx \frac{\varphi_G-\xi(\alpha)(\rho_1\rho_{G_2}+\rho_2\rho_{G_1})+\xi(\alpha)^2\rho_1\rho_2h_x^2}{p\sqrt{(1-\xi(\alpha)\rho_1^2)(1-\xi(\alpha)\rho_2^2)}}l_j+\frac{N_s\varphi'}{N_{1,sam}N_{2,sam}}.
\end{aligned} \tag{31}$$

The second approximation in Eq. (31) hides $\mathcal{O}(1/p)$ from disregarding the term $(\sum_{k=1}^p r_{kj}\beta_k)^2\xi(\alpha)$ in the denominator. Therefore,

$$\begin{aligned}
\mathbb{E}(\chi_{1j}\chi_{2j}) &= \sqrt{N_{1,sam}N_{2,sam}}\cdot\mathbb{E}(\gamma^*_{1j,sam}\gamma^*_{2j,sam}) \\
&\approx \frac{\frac{\sqrt{N_{1sam}N_{2sam}}}{p}\left[\varphi_G-\xi(\alpha)(\rho_1\rho_{G_2}+\rho_2\rho_{G_1})+\xi(\alpha)^2\rho_1\rho_2h_x^2\right]l_j+\frac{N_s\varphi'}{\sqrt{N_{1,sam}N_{2,sam}}}}{\sqrt{(1-\xi(\alpha)\rho_1^2)(1-\xi(\alpha)\rho_2^2)}}.
\end{aligned} \tag{32}$$

When $N_{1,sam}$ and $N_{2,sam}\to\infty$, the LDSC estimates of genetic covariance is

$$\mathbb{E}(\widehat{\varphi}_{G,LDSC}) = \frac{\varphi_G-\xi(\alpha)(\rho_1\rho_{G_2}+\rho_2\rho_{G_1})+\xi(\alpha)^2\rho_1\rho_2h_x^2}{\sqrt{(1-\xi(\alpha)\rho_1^2)(1-\xi(\alpha)\rho_2^2)}}. \tag{33}$$

E.3. Genetic covariance between participation and a related trait. Finally, we consider the LDSC estimate of the genetic correlation between participation and a related trait. As discussed in Section E.1, for the j-th SNP, we have the observed marginal effect in the sample of participants $\gamma^*_{j,sam}=\gamma'^*_j+\zeta_j$. The errors have $\mathbb{E}(\zeta_j)=0$; $\mathrm{Var}(\zeta_j)\approx 1/N_{sam}$ when $p\to\infty$. Note that we derive the estimated genetic effects of participation liability score, denoted as $\boldsymbol{\beta}^*_{IBD}$, based on IBD comparison among sibling pairs. Then we have $\beta^*_{j,IBD}=\beta^*_j+\eta_j$, where $\mathbb{E}(\eta_j)=0$; $\mathrm{Var}(\eta_j)\approx 1/N_{IBD}$ when $p\to\infty$, where N_{IBD} is the effective sample size in the IBD-based test. Specifically, the IBD-based analysis across sibling pairs and the GWAS study of other phenotypes based on unrelated samples have no sample overlaps. Then we have

$$\begin{aligned}
\mathbb{E}(\beta^*_{j,IBD}\gamma^*_{j,sam}|\boldsymbol{\beta},\boldsymbol{\gamma}) &= \mathbb{E}\left[(\beta^*_j\gamma'^*_j+\beta^*_j\cdot\zeta_j+\gamma'^*_j\cdot\eta_j+\eta_j\zeta_j)|\boldsymbol{\beta},\boldsymbol{\gamma}\right] \\
&= \beta^*_j\gamma'^*_j+\mathbb{E}(\eta_j\zeta_j)\approx\beta^*_j\gamma'^*_j.
\end{aligned} \tag{34}$$

From Equation Eq. (25), we have

$$\begin{aligned}
\mathbb{E}(\beta^*_{j,IBD}\gamma^*_{j,sam}) &= \mathbb{E}\left[\mathbb{E}(\beta^*_{j,IBD}\gamma^*_{j,sam}|\boldsymbol{\beta},\boldsymbol{\gamma})\right]\approx\mathbb{E}\left[\frac{\beta^*_j(\gamma^*_j-\xi(\alpha)\rho\beta^*_j)}{\sqrt{(1-\xi(\alpha)\rho^2)}\cdot(1-\xi(\alpha)\beta^{*2}_j)}\right] \\
&= \mathbb{E}\left\{\frac{(\sum_{k=1}^p r_{kj}\beta_j)\cdot\left[\sum_{k=1}^p r_{kj}(\gamma_k-\xi(\alpha)\rho\beta_k)\right]}{\sqrt{(1-\xi(\alpha)\rho^2)}\cdot\left[1-\xi(\alpha)(\sum_{k=1}^p r_{kj}\beta_k)^2\right]}\right\}\approx\frac{\rho_G-\xi(\alpha)\rho h_x^2}{p\sqrt{(1-\xi(\alpha)\rho^2)}}l_j.
\end{aligned} \tag{35}$$

The second approximation in Eq. (35) hides $\mathcal{O}(1/p)$ from disregarding the term $\xi(\alpha)(\sum_{k=1}^p r_{kj}\beta_k)^2$ in the denominator. Therefore,

$$\mathbb{E}(\chi_{j,x}\chi_j)=\sqrt{N_{IBD}N_{sam}}\cdot\mathbb{E}(\beta^*_{j,IBD}\gamma^*_{j,sam})\approx\frac{\frac{\sqrt{N_{IBD}N_{sam}}}{p}(\rho_G-\xi(\alpha)\rho h_x^2)}{\sqrt{(1-\xi(\alpha)\rho^2)}}l_j. \tag{36}$$

When $N\to\infty$, the LDSC estimates of genetic covariance between participation and the phenotype is

$$\mathbb{E}(\widehat{\rho}_{G,LDSC})=\frac{\rho_G-\xi(\alpha)\rho h_x^2}{\sqrt{1-\xi(\alpha)\rho^2}}. \tag{37}$$

F. Phenotypes. To generate the UKBB GWAS summary statistics, we follow the data process procedure in Benonisdottir and Kong (2023) (5), and adjusted the variables for year of birth, age at recruitment up to the order of three and 40 principal components. The resulting residuals for BMI, HGT, WC, HC, INC, WKT were then rank-based inverse normalised separately for each sex.

F.1. Body mass index (BMI). For the UKBB data, we constructed the BMI variable based on the data-field 21001-0-0: "Body Mass index (BMI)", which was derived from height and weight measurements recorded in the initial assessment visit. For the HSE data, we use the variable "bmival".

F.2. Height (HGT). For the UKBB data, we constructed the HGT variable based on the data-field 50-0-0: "Standing height", which was measured in the initial assessment visit. For the HSE data, we use the variable "htval".

F.3. Waist circumference (WC). For the UKBB data, we constructed the WC variable based on the data-field 48-0-0: "Waist circumference", which was measured in the initial assessment visit. For the HSE data, we use the variable "wstval".

F.4. Hip circumference (HC). For the UKBB data, we constructed the HC variable based on the data-field 49-0-0: "Hip circumference", which was measured in the initial assessment visit. For the HSE data, we use the variable "hipval".

F.5. Educational attainment (EA). For the UKBB data, We followed the construction of the EA variable in Benonisdottir and Kong (2023) (5), which was from the data-fields 6138 (Qualifications) and 845 (Age completed full-time education). Both data-fields contain answers to questions that participants were asked on a touch-screen in the initial assessment visit. Specifically, we map the degree to years of education:

- College or Universty degree: 20 years
- Other professional qualifications, *e.g.* nursing, teaching: 15 years
- A levels/AS levels or equivalent: 13 years
- O levels/GCSEs or equivalent: 10 years
- CSEs or equivalent: 10 years
- NVQ or HND or HNC or equivalent: (Age completed full time education -5) years
- None of the above: 7 years
- Prefer not to answer: excluded from the study

For the HSE data, we use the variable "quala", and map the degree to years of education:

- Degree/degree level qualification (including higher degree): 20 years
- Teaching qualification; Nursing qualifications SRN, SCM, SEN, RGN, RM, RHV, Midwife; HNC/HND, BEC/TEC Higher, BTEC Higher/SCOTECH Higher; City and Guilds Full Technological Certificate: 15 years
- ONC/OND/BEC/TEC/BTEC not higher; City and Guilds Advanced/Final Level; A-levels/Higher School Certificate; AS level; SLC/SCE/SUPE at Higher Grade or Certificate of Sixth Year Studies: 13 years
- City and Guilds Craft/Ordinary Level; O-level passes taken in 1975 or earlier; O-level passes taken after 1975 GRADES A-C; GCSE GRADES A-C; CSE GRADE 1/SCE BANDS A-C/Standard Grade LEVEL 1-3; SLC Lower; School Certificate or Matric; O-level passes taken after 1975 GRADES D-E; CSE GRADES 2-5/SCE Ordinary BANDS D-E; GCSE GRADES D-G; SUPE Lower or Ordinary; Recognised Trade Apprenticeship completed: 10 years
- NVQ Level 5; NVQ Level 4; NVQ Level 3/Advanced level GNVQ; NVQ Level 2/Intermediate level GNVQ; NVQ Level 1/Foundation level GNVQ: (Age completed full time education -5) years
- CSE Ungraded; Clerical or Commercial Qualification (*e.g.* typing/book-keeping/commerce): 7 years

F.6. Employment status (ES). For the UKBB data, we constructed ES variable with data-field 6142. The individuals answering "None of the above" or "Prefer not to answer" were excluded in the study. The other individuals were classified into "employed" (In paid employment or self-employed) and the others. For the HSE data, we used variable "econact", to classify individuals to "employed" (In employment) and the others. Those with answers "No answer/refused", "Don't know", "Refused/not obtained", "Schedule not obtained", "Schedule not applicable", and "Item not applicable" were removed from the study.

F.7. Income (INC). We used data-field 738 for the UKBB data, and variable "totinc" for the HSE data. We constructed the INC variable as an ordinal variable with average total household income 1: less than £18,000; 2: £18,000 to £31,000; 3: £31,000 to £52,000; 4: £52,000 to £100,000; 5: more than £100,000.

F.8. Current smoking status (SMC). We used data-field 20116 for the UKBB data, and variable "cignow" for the HSE data. The SMC is a binary variable indicating whether the individual smokes currently.

F.9. Previous smoking status (SMP). We used data-field 20160 for the UKBB data, and variable "smkevr" for the HSE data. The SMP is a binary variable indicating whether the individual has ever smoked.

F.10. Alcohol consumption (ALC). We used data-field 1558 for the UKBB data, and variable "dnoft" for the HSE data. We constructed the ALC variable as an ordinal variable with alchol intake frequency 1: never; 2: occasionally; 3: 1-3 times a month; 4: 1-2 times a week; 5: 3-4 times a week; 6: almost everyday.

F.11. Walking pace (WKP). We used data-field 924 for the UKBB data, and variable "walkpace" for the HSE data. We constructed the WKP variable as an ordinal variable with walking pace 1: slow; 2: average; 3: brisk. After adjusted for year of birth, age at recruitment, and sex, and top 40 principal components, the variable was rank-based inverse normal transformed, and treated as a continuous phenotype in heritability estimation.

F.12. Walking time (WKT). We used data-field 874 for the UKBB data, and variable "tottim" for the HSE data. The WKT variable measures duration of walk on a typical day by minutes.

F.13. Physical activity study invitation. For the UKBB data, we use the physical activity study invitation variable based on data-field 110005: "Invitation to physical activity study, acceptance". The values "No response", "Partial", or "Completed" were defined as cases (*i.e.* those had been invited to participate the study). The individuals with non-applicable (NA) values were defined as controls (*i.e.* those had not been invited to the study.)

F.14. Physical activity study participation. For the UKBB data, we use the physical activity study participation variable based on data-field 110005: "Invitation to physical activity study, acceptance". The values "Partial" or "Completed" were defined as cases, and the values "No response" were defined as controls.

G. A note on IPW adjustment. Schoeler et al. (2023) proposed an IPW adjustment for participation bias (6). For this IPW adjustment to be sufficient for analyses that include genotypes, it requires that the genetic component underlying participation manifests its effect entirely through the propensity score. We show this assumption is problematic by performing association analysis for polygenic risk score of participation constructed with IBD-based test statistics (denoted as PGS) and another phenotype, adjusting for the predicted participation. In parallel to Schoeler et al. (2023), we included 14 harmonized characteristics between UKBB and HSE, *i.e.* frequency of alcohol (categorical), sex (binary), age (continuous), years of education (categorical), smoking status (categorical), income (categorical), household size (categorical), employment status (categorical), height (continuous), weight (continuous), BMI (continuous), BMI (categorical), urbanisation (categorical), and overall health (categorical). All categorical and binary variables were entered as dummy variables, and we used LASSO regression to predict UKBB participation (UKBB=1; HSE=0). The tuning parameter for LASSO regression was tuned with five-fold cross-validation that minimizes the cross-validated error. The predicted probabilities were used to construct the propensity scores.

We considered four phenotypes that are significantly associated with the pPGS: EA, BMI, and the invitation and participation in a physical activity study. Specifically, we performed a linear/logit regression with a phenotype as the response, and including the pPGS and the propensity score as covariates. When adjusting for the propensity score, the associations between the pPGS and the four variables shrunk by some degree, but remained highly statistically significant (p ranges from 1.7×10^{-5} to $< 2 \times 10^{-16}$ (Table S2), indicating that the assumption does not hold.

We also demonstrate these issues via simulation studies. First, consistent to the simulation settings we described in the main text, we simulated genetic data $\boldsymbol{G}$ for a population of 5×10^5 individuals. Then for each time of the simulations, we simulated $\boldsymbol{\beta}$, $\boldsymbol{\gamma}_1$, and $\boldsymbol{\gamma}_2$ with multivariate normal distribution, and generated the participation liability score for each sample with $X = \boldsymbol{G\beta} + \epsilon_x$, and other two phenotypes $Y_1 = \boldsymbol{G\gamma}_1 + \epsilon_{y_1}$, and $Y_2 = \boldsymbol{G\gamma}_2 + \epsilon_{y_2}$. As described in the main text, the genetic and non-genetic correlations between Y_1, Y_2, and X were varied. The participation rate (α) was set to 0.055, which matches the participation rate of the UKBB. Samples with $X > t_\alpha$ were selected as participants, which leads to 275,000 participants. In order to perform IPW described in Schoeler et al. (2023), we randomly sampled 20,000 people in the total population as a "representative" population, which match the sample sizes of HSE data used as "control" in Schoeler et al. (2023). We then followed their procedure to predict participation with a logistic regression model, with $N = 275,000$ participants as cases and the 20,000 randomly sampled individuals as controls, with Y_1 and Y_2 as predictors. The weights for each sample are generated by $w = (1-p)/p$, where p is the predicted probability of participating. We also followed Schoeler et al. (2023) to compute the effective sample size as $N_{eff} = N/(Var(w_{in}) + 1)$, where w_{in} is the normalized weights across study participants. As shown in Table S1, the IPW method yielded greater bias in both heritability and genetic correlation estimates in all simulation settings.

H. Multi-ancestry analysis in the UKBB. Our analysis on the main text focuses on the white British (WB) samples in the UKBB. Here we provide some results for other ancestries. We first trained a random forest classification model using the first 20 ancestry principal components and corresponding continental population labels in the 1000 Genomes Project (7, 8), including European ancestry (EUR), African ancestry (AFR), American ancestry (AMR), South Asian ancestry (SAS), and East Asian ancestry (EAS).

For each ancestry group, we computed the correlations between EA and the participation polygenic risk scores ($pPGS$) based on genetic effects estimated in WB samples, across different ancestries in the UKBB (Table S4). The correlation between EA and $pPGS$ for the non-WB samples of European Ancestry (EUR) ($n = 53,090$) is not only highly statistically significant, but also comparable with that computed in the WB samples (0.032 versus 0.031). This suggests that the participation behavior of WB and non-WB EUR is very similar both phenotypically and genetically, although it is not a definitive proof. For other ancestries, the sample sizes are much smaller and only the correlation in the South Asians (SAS, $n = 10,107$) is nominally significant ($p = 0.048$). The standard errors are however so large that not much can be said about these other groups. For comparison, we also show the results for the correlation between EA and an EA polygenic score constructed using genetic effects estimated from the WB samples, across different ancestries. The correlation in the non-EUR WB is slightly smaller than the correlation in the WB samples, but not by much (0.255 versus 0.278). The correlations in the other groups are all

statistically significant but the magnitudes are substantially lower. We hypothesize that the participation genetic component might exhibit a similar trend, which remains for future research.

Table S1. Theoretical and simulation results for heritability and genetic correlation estimates. Standard deviations of 50 repeated simulations are shown in brackets. The heritability of participation liability score is fixed at 0.125. h^2_y, ρ_g, and φ_g are the population-level simulation settings; $h^2_{y,PB}$ and $\varphi_{g,PB}$ are the theoretical results in the sample; $\widehat{h}^2_y$ and $\widehat{\varphi}_g$ are the estimates in the sample; $\widetilde{h}^2_y$ and $\widetilde{\varphi}_g$ are the adjusted values; $\widetilde{h}^2_{y,IPW}$ and $\widetilde{\varphi}_{g,IPW}$ are the adjusted values with IPW procedure.

h^2_y (True)	ρ_g	ρ_e	$h^2_{y,PB}$	$\widehat{h}^2_y$	$\widetilde{h}^2_y$	$\widetilde{h}^2_{y,IPW}$
0.2	0.25	0.5	0.230	0.229 (0.009)	**0.198 (0.011)**	0.237 (0.011)
0.2	0	0.5	0.257	0.254 (0.009)	**0.198 (0.011)**	0.274 (0.011)
0.2	0.4	0.1	0.192	0.189 (0.008)	**0.198 (0.012)**	0.178 (0.007)
0.5	0.25	0.5	0.545	0.549 (0.014)	**0.501 (0.016)**	0.484 (0.015)
0.5	0	0.5	0.564	0.568 (0.013)	**0.503 (0.017)**	0.566 (0.016)
0.5	0.4	0.1	0.492	0.490 (0.013)	**0.494 (0.019)**	0.370 (0.015)

(a) Theoretical and simulation results for the heritability.

φ_g (True)	ρ_{g_1}	ρ_{g_2}	$\varphi_{g,PB}$	$\widehat{\varphi}_g$	$\widetilde{\varphi}_g$	$\widetilde{\varphi}_{g,IPW}$
0.5	0.25	0.25	0.458	0.459 (0.024)	**0.496 (0.030)**	0.607 (0.023)
0.5	0.25	0	0.472	0.472 (0.025)	**0.502 (0.029)**	0.681 (0.019)
0.5	0	0	0.516	0.515 (0.023)	**0.498 (0.030)**	0.678 (0.018)
0.2	0.25	0.25	0.140	0.143 (0.029)	**0.196 (0.035)**	0.220 (0.038)
0.2	0.25	0	0.175	0.178 (0.030)	**0.202 (0.037)**	0.353 (0.034)
0.2	0	0	0.232	0.232 (0.028)	**0.199 (0.038)**	0.387 (0.031)

(b) Theoretical and simulation results for the genetic correlation between two phenotypes other than participation. The heritabilities of the two phenotypes were set to 0.5 and 0.2. The non-genetic correlation with participation (ρ_e) was fixed at 0.5.

Table S2. The pPGSs association with phenotypes adjusted for propensity scores of participation. The propensity scores were constructed by comparing 14 harmonized characteristics of UKBB participants with HSE. Linear regression was used for analyzing EA and BMI, and logistic regression was used for the two secondary invitation and participation for physical activity study. Displayed are fitted coefficients (Effect/log(OR)) and p-values from regressing phenotypes on the pPGS in a subset of White British unrelateds, taking sex, year of birth, age at recruitment, and 40 principal components as covariates. The pPGS and the quantitative phenotypes were transformed to have a variance of 1 and thus effect is in s.d. units.

Phenotypes	pPGS			pPGS adjusted for predicted participation		
	Effect	p-value	Sample size	Effect	p-value	Sample size
EA	3.1×10^{-2}	$< 2 \times 10^{-16}$	268,205	2.0×10^{-2}	$< 2 \times 10^{-16}$	227,688
BMI	-1.9×10^{-2}	$< 2 \times 10^{-16}$	270,330	-1.6×10^{-2}	8.9×10^{-15}	227,688
Secondary invitation	2.7×10^{-2}	3.5×10^{-12}	132,148/139,048	2.1×10^{-2}	9.0×10^{-7}	114,834/112,854
Secondary participation	3.0×10^{-2}	7.6×10^{-8}	59,264/72,884	2.6×10^{-2}	1.7×10^{-5}	52,317/62,517

Table S3. Estimated heritability with full 402,377 WB samples and with 337,208 unrelated WB individuals. The larger estimates are highlighted in boldface.

Phenotype	Estimated using full 402,377 WB samples	Estimated using 337,208 unrelated WB individuals
BMI	0.253 (0.011)	0.253 (0.011)
HGT	0.545 (0.026)	**0.546 (0.026)**
WC	0.211 (0.010)	0.211 (0.010)
HC	0.235 (0.011)	**0.236 (0.011)**
EA	0.211 (0.006)	0.211 (0.006)
ES	**0.020 (0.002)**	0.018 (0.003)
INC	0.083 (0.004)	0.083 (0.004)
SMC	**0.135 (0.008)**	0.131 (0.008)
SMP	0.115 (0.004)	0.116 (0.005)
ALC	**0.084 (0.004)**	0.081 (0.004)
WKP	0.077 (0.003)	0.077 (0.003)
WKT	**0.043 (0.002)**	0.042 (0.002)

Table S4. Polygenic risk scores (PGS) of participation and EA and their associations with educational attainment (EA) across different ancestry groups. Linear regression was used for analyzing EA and PGSs. Displayed are fitted coefficients and p-values from regressing EA on the $pPGS$ and PGS_{EA}, taking sex, year of birth, age at recruitment, and 40 principal components as covariates. The $pPGS$, PGS_{EA}, and EA were transformed to have a variance of 1 and thus effects in s.d. units. Standard errors are shown in brackets. Samples sizes are shown for White British (WB), European ancestry excluding WB (EUR, no WB), African ancestry (AFR), American ancestry (AMR), South Asian ancestry (SAS), and East Asian ancestry (EAS). The participation PGS ($pPGS$) were calculated based on genetic effects estimated from IBD-based information in the WB samples. The EA PGS (PGS_{EA}) were calculated based on genetic effects estimated from $272,425$ unrelated WB samples. Significant associations ($p < 0.05$) are highlighted in boldface.

Ancestry	Sample size	$pPGS$		PGS_{EA}	
		Effect	p-value	Effect	p-value
WB	402,302	**0.031 (0.002)[a]**	$< 2.2 \times 10^{-16}$	**0.278 (0.003)[b]**	$< 2.2 \times 10^{-16}$
EUR (no WB)	53,090	**0.032 (0.005)**	5.1×10^{-12}	**0.255 (0.004)**	$< 2.2 \times 10^{-16}$
AFR	9,482	-0.022 (0.021)	0.300	**0.083 (0.011)**	2.7×10^{-15}
AMR	1,764	0.044 (0.029)	0.121	**0.170 (0.024)**	1.9×10^{-12}
SAS	10,107	**0.022 (0.011)**	0.048	**0.161 (0.010)**	$< 2.2 \times 10^{-16}$
EAS	2,694	0.003 (0.021)	0.881	**0.098 (0.020)**	1.3×10^{-6}

[a] 272,425 unrelated WB samples were tested to avoid overfitting.
[b] 129,877 related WB samples were tested to avoid overfitting.

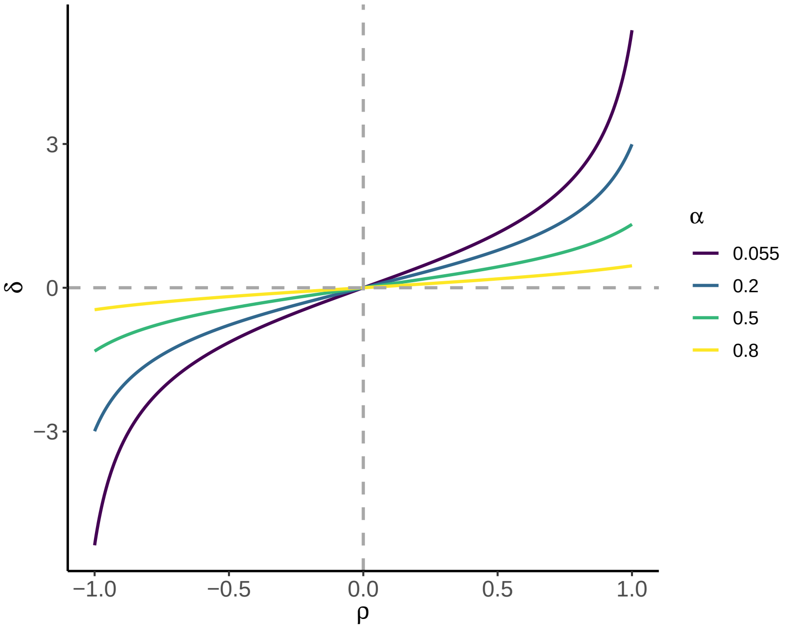

Fig. S1. The relationship between mean shifts (δ) and phenotypic correlation between X and Y (ρ), under varying participation rates (α).

References

1. R Kan, C Robotti, On moments of folded and truncated multivariate normal distributions. *J. Comput. Graph. Stat.* **26**, 930–934 (2017).
2. BK Bulik-Sullivan, et al., LD score regression distinguishes confounding from polygenicity in genome-wide association studies. *Nat. Genet.* **47**, 291–295 (2015).
3. DA Harville, Approximating the selection process. *Biometrics* **26**, 51–66 (1970).
4. J Jiang, W Jiang, D Paul, Y Zhang, H Zhao, High-dimensional asymptotic behavior of inference based on GWAS summary statistics. *Stat. Sinca* **3**, 1555–1576 (2023).
5. S Benonisdottir, A Kong, Studying the genetics of participation using footprints left on the ascertained genotypes. *Nat. Genet.* **55**, 1413–1420 (2023).
6. T Schoeler, et al., Participation bias in the UK Biobank distorts genetic associations and downstream analyses. *Nat. Hum. Behav.* **7**, 1216–1227 (2023).
7. GP Consortium, , et al., A global reference for human genetic variation. *Nature* **526**, 68 (2015).
8. T Chen, H Zhang, R Mazumder, X Lin, Splendid incorporates continuous genetic ancestry in biobank-scale data to improve polygenic risk prediction across diverse populations. *bioRxiv* pp. 2024–10 (2024).
